# Higher levels of B‐cell mutation in the early germinal centres of an inefficient secondary antibody response to a variant influenza haemagglutinin

**DOI:** 10.1111/imm.13052

**Published:** 2019-03-11

**Authors:** Richard K. Tennant, Barbara Holzer, John Love, Elma Tchilian, Harry N. White

**Affiliations:** ^1^ Department of Biosciences University of Exeter Exeter UK; ^2^ The Pirbright Institute Pirbright UK

**Keywords:** affinity maturation, antibodies, B‐cell memory, cross‐reactive, influenza virus

## Abstract

Designing improved vaccines against mutable viruses such as dengue and influenza would be helped by a better understanding of how the B‐cell memory compartment responds to variant antigens. Towards this we have recently shown, after secondary immunization of mice with a widely variant dengue virus envelope protein with only 63% amino acid identity, that IgM^+^ memory B cells with few mutations supported an efficient secondary germinal centre (GC) and serum response, superior to a primary response to the same protein. Here, further investigation of memory responses to variant proteins, using more closely related influenza virus haemagglutinins (HA) that were 82% identical, produced a variant‐induced boost response in the GC dominated by highly mutated B cells that failed, not efficiently improving serum avidity even in the presence of extra adjuvant, and that was worse than a primary response. This supports a hypothesis that over a certain level of antigenic differences, cross‐reactive memory B‐cell populations have reduced competency for affinity maturation. Combined with our previous observations, these findings also provide new parameters of success and failure in antibody memory responses.

**Abbreviations**
Bris: Brisbane 59/07
ELISA: enzyme‐linked immunosorbent assay
GC: germinal centre
HA: haemagglutinin
RT‐PCR: reverse transcription–polymerase chain reaction
SHM: somatic hypermutation

## Introduction

Foreign antigens stimulate the formation of memory B cells that have undergone somatic hypermutation (SHM) of their antibody genes and selection in germinal centres (GCs).^1^, ^2^ In this manner, immunity is established against identical pathogens. Despite the importance of understanding protection against mutable viruses such as dengue and influenza viruses, however, how memory B cells respond to variant antigens is poorly understood.

We have recently shown that antibody memory responses to widely variant proteins involved secondary GCs with a higher proportion of IgM^+^ B cells, with fewer VH mutations,^1^ compared with the memory response to the homotypic antigen. These observations support the idea that ‘lower’ layers of the B‐cell memory compartment, with less SHM, could furnish secondary responses against variant antigens.^3^, ^4^, ^5^

A complication of sequential infection by certain variant viruses, however, is that immune responses to the second virus can be compromised. In such situations, termed antigenic sin, cross‐reactive memory‐derived antibodies are induced that have a lower avidity and may increase pathology, as they are non‐neutralizing or enhance infectivity.^6^, ^7^, ^8^, ^9^ A critical question is why such memory responses then fail to rapidly evolve higher affinity to variant antigens through a secondary GC reaction.

Using sequential immunization with variant influenza virus haemagglutinins (HA), which we have selected because they are more closely related than the previously used dengue virus proteins, we have identified an antibody memory response to a variant antigen that exhibits inefficient secondary affinity maturation associated with altered GC reactions and mutation profiles, linking ‘antigenic sin’ to altered GC activity for the first time.

## Materials and methods

### Mice, immunization, antigens

All antigens were from Sino Biologicals (Beijing, China) and female 8‐ to 11‐week‐old BALB/c mice were obtained from Charles River Laboratories (Margate, UK). Most primary immunizations were, 25 μg PR8/34 HA1 alum precipitated with 2 × 10^7^ heat‐killed *Bordetella pertussis* administered intraperitoneally. Brisbane 59/07 (Bris) HA1 or HA was also used for priming as stated in the Results section, also with alum/pertussis. Secondary immunizations were 25 μg PR8/34 or Bris HA protein administered intraperitoneally in phosphate‐buffered saline, or with Sigma Adjuvant System (Sigma‐Aldrich, Gillingham, Dorset, UK), as stated in the Results section. Rationale for HA1 priming/HA boost was to focus the response away from the conserved stem and on to the variable epitopes of the head and to avoid sequential exposure to the C‐terminal HA1/His tag region, to reduce confounding sequence similarities. All animal experiments were performed under UK Home Office license PPL 30/3089 with permission from the University of Exeter local animal welfare ethics review board.

### ELISA

Standard protocol for enzyme‐linked immunosorbent assay (ELISA) used 1 μg/ml coating protein in bicarbonate buffer. End‐point titre values plotted are log_2_ of 1/(end‐point dilution × 100), each unit increase represents a doubling of titre.

### Urea avidity ELISA

Standard ELISA was performed, following Puschnik *et al*.,^10^ then after the serum step, we performed 1× wash, 10‐min incubation with 7 m urea/Phosphate‐buffered saline Tween (PBST), 2× wash, then standard protocol. Avidity index was calculated as 7‐m urea readings/readings from PBST‐only treatment, after subtraction of background.

### Flow cytometry

Red blood cell‐depleted spleen cells were incubated with Fc‐block (BD Biosciences, Wokingham, Berkshire, UK) then allophycocyanin‐conjugated anti‐B220, BV421‐conjugated anti‐CD38 and phycoerythrin‐conjugated anti‐CD95/Fas (BD), and anti‐IgM (eBioscience/Thermo‐Fisher Scientific, Paisley, UK), following standard protocols.

### B‐cell antibody sequencing

Single GC B cells were sorted into 10 μl chilled 10 mm Tris–HCl pH 8·0, 1 U/μl RNAsin (Promega, Southampton, UK) and frozen at −80°. One‐Step reverse transcription–polymerase chain reaction (RT‐PCR) (Qiagen, Manchester, UK) was performed with primer sets as described by Tiller *et al*.^11^, for 50 cycles annealing at 53·6°. Heavy‐chain second‐round PCRs used 2 μl first‐round PCR, Tiller *et al*.^11^ primers, and *Taq* (Qiagen) for 50 cycles with annealing at 56°. Products were sequenced using primer 5ʹ‐MsVHE,^11^ which leaves part of the 5ʹ end of FR1 unsequenced; hence, FR1 was not analysed. VH sequences were analysed using IMGT V‐quest platform. Sequences are given in the Supplementary material (Table S1).

### Statistics

Three mice were randomly allocated to cages. For greater sample sizes, treatments were independently replicated. Where *t*‐test was applied, data points were analysed for equality of variance; where this was violated they were subject to a two‐tailed test for unequal variance, otherwise a two‐tailed test for equal variance was used.

## Results

### Primary and secondary responses to PR8 HA

We defined the serum and GC response after a homotypic antigen prime‐boost. Mice were primed with PR8/34 (PR8) HA1 with adjuvant and boosted 38 days later with soluble PR8 HA (see Materials and methods for explanation).

**Figure 1:**
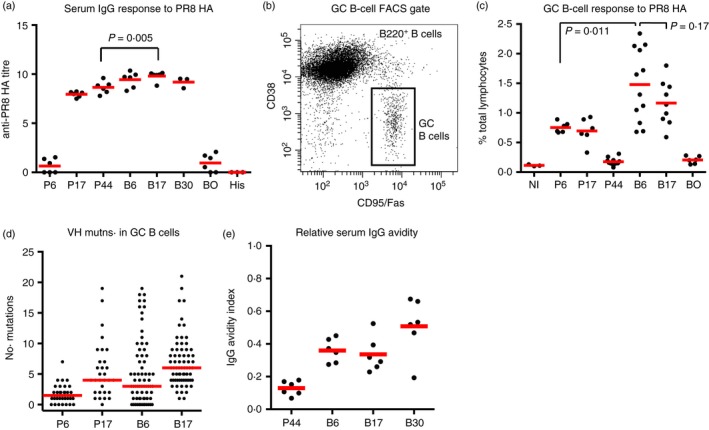
Primary and secondary response to homotypic PR8 haemagglutinin (HA). (a) Anti‐PR8 HA serum IgG response. Bar shows mean. P*x*,* x* days since priming; B*x*,* x* days since boosting; BO, adjuvant‐only primed, PR8 HA boosted, analysed day 6; His, B6 serum sample reactivity with irrelevant C‐terminal His‐tagged protein (*Bacillus clausii* pdxR). (b) FACs gating strategy. (c) GC B‐cell response to PR8 HA. Bar shows mean. NI, not immunized; other labels as for panel (a). (d) VH mutations in single sorted germinal centre (GC) B cells. From *n* = 3 mice (P*x*) and *n* = 6 mice (B*x*). Bar shows median value. Labels as for panel (a). (e) Relative serum IgG avidity for PR8 HA. Bar shows mean. Labels as for panel (a).

Priming induced an increasing IgG titre to day 44 (Fig. 1a). Boosting increased the titre modestly (twofold) but significantly by day 17. Levels of GC B cells rose from a background of 0·18% to 0·75% by day 6 after priming, remained elevated until day 17, and fell to background by day 44 (Fig. 1b,c). Boosting induced GC B cells to a significantly higher level, 1·5%, by day 6, which reduced by day 17, but not significantly (Fig. 1c).

VH mutations increased by day 17 after priming, consistent with active SHM and affinity maturation (Fig. 1d). Boosting induced GC B cells with a higher level of VH mutation at day 6 (median = 3) than seen at day 6 after priming (median = 1·5), consistent with a response from memory B cells focused on the HA1 head region. GC B cells then accumulated further mutations by day 17 after boost (median = 6), consistent with further affinity maturation. Analysis of the relative avidity of serum IgG (Fig. 1e) showed a rapid increase by day 6 after PR8 HA boosting, which points to stimulation of the highest affinity memory cells by the homotypic boost. There was a further increase by day 30, up to 51%.

### A failed secondary response to heterotypic HA

**Figure 2:**
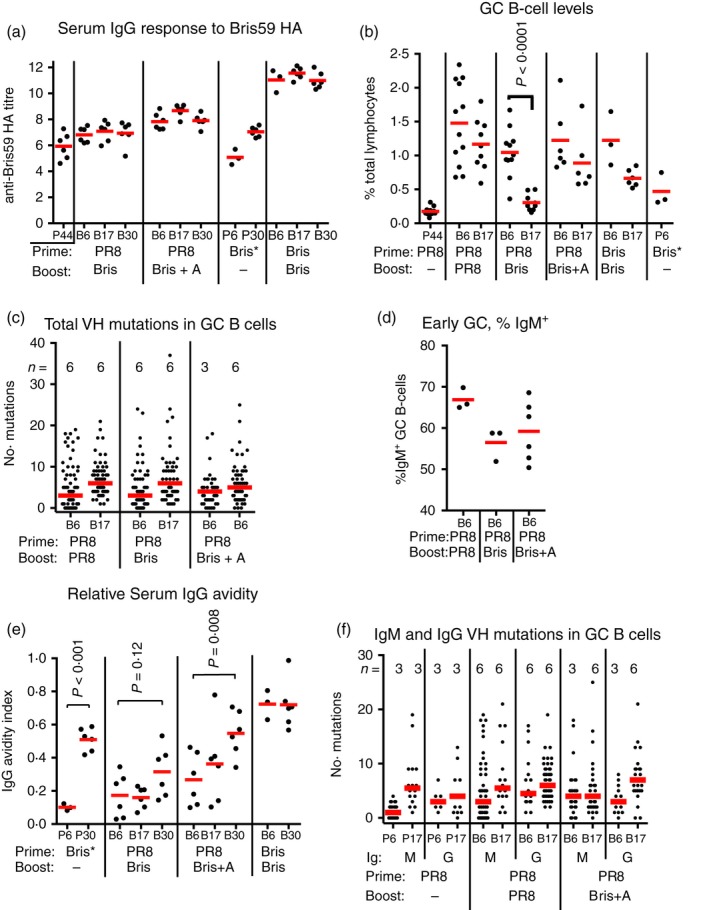
Secondary responses to variant haemagglutinin (HA) boosting. (a) Anti‐Brisbane 59/07 (Bris) HA serum IgG titres after Bris HA boosting. Bars indicate mean. *x*‐axis labels: P44, PR8 HA1 prime only, day 44. All other labels: P*x*,* x* days since priming with antigen indicated below; B*x*,* x* days since boosting with antigen indicated below. Bris, Bris HA, Bris + A, Bris HA including adjuvant. Note: The Bris* prime was performed with Bris HA, not HA1, to allow appropriate comparison with Bris HA‐boosted groups. (b) Germinal centre (GC) B‐cell response to Bris HA boost. Bars show mean. First three data sets reproduced from Fig. 1(c) for comparison. *x*‐axis labels as for panel (a). (c) VH mutations in single sorted GC B cells. From *n* = mice in each group indicated. Bar shows median. *x*‐axis labels as for panel (a). First two data sets reproduced from Fig. 1(d) for comparison. (d) Proportion of IgM^+^ GC B‐cells in day 6 GC. Bar shows mean. *x*‐axis labels as for panel (a). (e) Relative serum IgG avidity for Bris HA. Bars show mean. *x*‐axis labels as for panel (a). (f) VH mutations in IgM^+^ and IgG^+^ GC B cells. Number of mice in each group indicated as *n* = . Bars indicate median. *x*‐axis labels as for panel (a).

After PR8 HA1 priming, we performed boosts with Bris HA, which has 82% identity with PR8 over the HA1 region. Initially, the boosts were not adjuvanted because this was not previously necessary to induce successful heterotypic secondary responses.^1^ In the experiments reported below, however, because of the poor durability of the non‐adjuvanted boost response in the GC, we repeated all boosts with an adjuvant. In this way, we sought also to determine if adjuvant increased the longevity of the GC response, and if it did, what effect this had on the resulting affinity maturation. Figure 2(a) shows the serum titres induced by the heterotypic Bris HA boosts, a Bris HA prime and a Bris/Bris prime‐boost.

Without adjuvant, the heterotypic Bris HA boost induced a robust GC response by day 6, up to just over 1·0%, which collapsed by day 17 to 0·3%, close to the pre‐boost background level of 0·18% (Fig. 2b). This contrasted with the sustained response seen after PR8 HA boosting, and the responses after boosting with heterotypic dengue virus proteins, which were still fourfold to eightfold above background at day 17.^1^ Analysis of total VH mutations in Bris HA boosted GC B cells showed the same profile as the PR8 HA boost (Fig. 2c). With the previously reported heterotypic dengue virus proteins, early GC B cells had fewer mutations, compared with the homotypic boost.^1^ Further, here, at day 6 after Bris HA boosting, there was a lower proportion of IgM^+^ B cells (Fig. 2d) compared with the homotypic PR8 boost, again contrasting with the dengue virus protein responses. There was not a significant increase in the relative avidity of serum IgG detected by day 30 after Bris HA boost, to 32% (Fig. 2e). This rise was less than that seen after a Bris HA prime, up to 51% at day 30 (Fig. 2e), and heterotypic dengue‐4 envelope‐protein boosting, up to 53% by day 32.^1^

To test whether Bris HA is not just a weaker antigen, we performed a homotypic Bris/Bris prime‐boost. This induced similar levels of early GC B cells (1·2%; Fig. 2b) to the homotypic PR8 prime‐boost (1·5%; Figs 1c and 2b), which then declined slightly by day 17, to 0·7%. It also indeced twofold higher serum IgG titres (Fig. 2a) compared with the homotypic PR8 prime‐boost (Fig. 1a), suggesting that its antigenicity is similar.

### Effect of adjuvant on the heterotypic Bris HA boost response

Adjuvants containing Toll‐like receptor ligands can increase GC responses.^12^

Adjuvanted Bris HA boosts induced a nearly fourfold increase in IgG titre at day 17, above that of the non‐adjuvant boost (Fig. 2a), and induced sustained GC B‐cell levels, rising to 1·2% of lymphocytes by day 6 and only falling to 0·9% by day 17 (Fig. 2b). At day 30, however, the IgG titre was similar in adjuvanted Bris HA‐boosted mice compared with Bris HA‐primed mice (Fig. 2a).

Early GC B cells had similar, if not greater, levels of VH mutation (median = 4) compared with the non‐adjuvanted boost (median = 3; Fig. 2c), and an equivalent proportion of IgM GC B cells (Fig. 2d), implying that no extra naive cells were recruited to GCs by adjuvant. By day 17, median VH mutations in this group had only increased by one (Fig. 2c).

The adjuvanted Bris HA boost induced a significant increase in serum avidity from day 6 to day 30, but only to levels comparable to those seen with the Bris HA prime group, being 54% versus 51%, and not approaching the avidity of the homotypic Bris HA1/Bris HA prime‐boost which was 72% (Fig. 2e). Considering the net level of increase in avidity between day 6 and day 30, the Bris HA prime was more efficient (41% increase) than the adjuvanted heterotypic boost (28% increase).

### Altered selection of VH mutations in Bris HA‐boosted mice

Despite a sustained GC reaction, the adjuvanted Bris HA response only supported a slight increase in overall VH mutation (median = +1) between days 6 and 17 (Fig. 2c), compared with the PR8 HA1 primary (+2·5), PR8 HA boost (+3) and dengue‐4 envelope protein boost (+4).^1^ Analysis of the levels of VH mutations in IgM and IgG showed further evidence of altered selection. The primary and secondary responses to PR8 HA and the heterotypic dengue protein E4 and E2 responses all showed increases in IgM VH mutation of between +1·5 and +4·5 as the GC response matured (Fig. 2f, and ref. 1). In the adjuvanted Bris HA boost response, there was no increase in IgM mutations. The non‐adjuvanted Bris HA boost response was not included in this analysis as the failure of the GC reaction implies that few cells were successfully selected to day 17.

## Discussion

We have shown how B‐cell memory responses to boosting with heterotypic antigens can be inefficient. With the non‐adjuvanted heterotypic Bris HA boosts, GC B‐cell numbers collapsed by day 17 despite ongoing SHM, and serum avidity failed to increase significantly, contrasting with heterotypic dengue protein boosts in mice, and reproducing an ‘antigenic sin’ response. With adjuvant, Bris HA boosting induced comparable IgG titres but a smaller increase in serum avidity than the primary response to Bris HA. Considering the adjuvanted Bris HA boost response initiated with 3·6‐fold higher numbers of GC B cells (day 6) above background (Fig. 2b), it is less efficient than the primary response. The presence of a cross‐reactive memory B‐cell compartment, therefore, was an impediment to the response to Bris HA.

We do not consider that the failed secondary response to the non‐adjuvanted heterotypic Bris HA boost could be due to blocking by pre‐existing cross‐reactive antibodies because this effect would be stronger in the homotypic boosts (PR8/PR8 and Bris/Bris), which both show longevity of the GC reaction.

We previously reported that successful heterotypic secondary responses had early GCs containing more IgM^+^ B cells with fewer mutations.^1^ The heterotypic responses to Bris HA reported here had early GCs with lower proportions of IgM^+^ B cells (Fig. 2d), with equivalent or even higher levels of mutation compared with the homotypic boost (Fig. 2c). In the adjuvanted Bris HA boost, the high levels of mutation in IgM failed to increase further by day 17 (Fig. 2f). We propose that these heterotypic secondary responses to Bris HA were compromised because the antibody's ability to evolve was reduced. This could be due to an intrinsic loss of ability to evolve in mutated V genes, as they have lost hotspots for the *aicd* mutator, lost codons predisposed to non‐conservative substitutions^13^ and despite being diverse at the sequence level may be more restricted in V‐gene diversity. It could also be for GC dynamic reasons, such as increased SHM and class switching affecting GC B‐cell longevity and fate.^14^ The model of Deem and Lee^15^ provides a mathematical description of this. It predicts that over a certain window of antigenic difference, highly mutated memory responses to variant antigens can be worse than primary responses, despite memory B cells initiating with a higher affinity than naive cells, which also explains their dominance of the recall response.

It seems that the HA1 region of the Bris HA protein was sufficiently similar to PR8 HA1 (82% identity) to recruit the most mutated, and more often class‐switched, anti‐PR8 HA1 memory cells, which were then inefficient at further improving affinity for Bris HA. This outcome contrasts with the efficient affinity maturation previously observed with less mutated memory B cells^1^ and indicates that mutation levels are an important parameter for assessing the eventual success of cross‐reactive vaccine protection. We consider that the effects reported here are relevant to understanding sub‐optimal immune responses against pandemic strains of influenza where antigenic differences are larger compared with seasonal influenza epidemics, which are usually associated with a few mutations in the HA protein.

## Disclosures

The authors declare no conflicts of interest.

## Supporting information


**Table S1.** VH sequences excluding FR1
